# Hyperglycemia, hydroxychloroquine, and the COVID‐19 pandemic

**DOI:** 10.1002/jmv.25887

**Published:** 2020-04-27

**Authors:** Adam Brufsky

**Affiliations:** ^1^ UPMC Hillman Cancer Center, Magee Women's Hospital University of Pittsburgh School of Medicine Pittsburgh Pennsylvania

**Keywords:** antibody‐mediated cell‐mediated cytotoxicity, antiviral agents, SARS coronavirus

## Abstract

Coronavirus disease‐2019 (COVID‐19) infection and its severity can be explained by the concentration of glycosylated severe acute respiratory syndrome‐coronavirus 2 (SARS‐CoV‐2) viral particles in the lung epithelium, the concentration of glycosylated angiotensin‐converting enzyme receptor 2 (ACE2) in the lung epithelium, and the degree and control of the pulmonary immune response to the SARS‐CoV‐2 spike protein at approximately day 8 to 10 after symptom onset, which may be related to both. Binding of ACE2 by SARS‐CoV‐2 in COVID‐19 also suggests that prolonged uncontrolled hyperglycemia, and not just a history of diabetes mellitus, may be important in the pathogenesis of the disease. It is tempting to consider that the same mechanism acts in COVID‐19 as in SARS, where an overactive macrophage M1 inflammatory response, as neutralizing antibodies to the SARS‐CoV‐2 spike protein form at day 7 to 10, results in acute respiratory distress syndrome (ARDS) in susceptible patients. It also allows consideration of agents, such as hydroxychloroquine, which may interfere with this overly brisk macrophage inflammatory response and perhaps influence the course of the disease, in particular, those that blunt but do not completely abrogate the M1 to M2 balance in macrophage polarization, as well as viral load, which in SARS appears to be temporally related to the onset of ARDS.

## Highlights

(1) Aberrant glycosylation of SARS‐CoV‐2, ACE2, and various immunoregulatory proteins such as the Fc gamma receptor may impact the severity of COVID‐19 disease.
(2) Hyperglycemia, and not simply diabetes, may increase glycosylation of these proteins and lead to more severe COVID‐19 disease.
(3) Agents that block glycosylation may blunt the severity of COVID‐19.

## 1 ROLE OF THE ACE2 RECEPTOR IN COVID‐19 INFECTION

We are all struggling to understand the human catastrophe of the coronavirus disease‐2019 (COVID‐19) epidemic. As of April 12, 2020, there were 557043 cases of documented COVID‐19 infection in the United States, and 21952 deaths.^1^ Our economy except for limited sectors has come to a complete halt as we practice physical distancing to try to mitigate the effects of the pandemic.

In the 10 weeks since COVID‐19 began to accelerate, there has been a flurry of information from corners expected and unexpected to help us with this understanding. Rapid publication of peer‐reviewed data has defined the possible risk factors for COVID‐19, its clinical course, and its possible epidemiology. In this unusual time of a public health emergency, numerous non‐peer‐reviewed manuscripts have been uploaded to preprint servers, and their unreviewed data and conclusions must be evaluated in this spirit.

Nevertheless, data both published and in preprint form point to a tantalizing hypothesis: that COVID‐19 infection and its severity can be explained by the concentration of glycosylated severe acute respiratory syndrome‐coronavirus 2 (SARS‐CoV‐2) viral particles in the lung epithelium, the concentration of glycosylated angiotensin‐converting enzyme receptor 2 (ACE2) in the lung epithelium, and the degree and control of the pulmonary immune response to the SARS‐CoV‐2 spike protein at approximately day 8 to 10 after symptom onset, which may be related to both.

ACE2 appears to be the primary receptor for entry of SARS‐CoV‐2 into various epithelial tissues. An envelope anchored spike protein allows entry of coronaviruses into host cells by binding to a host receptor and fusing viral and host membranes.^2^ Analysis of SARS‐CoV, a betacoronavirus most similar to SARS‐CoV‐2 and responsible for Severe Acute Respiratory Syndrome (SARS),^3^ defined a receptor‐binding domain (RBD) of the SARS‐CoV spike protein that specifically recognized ACE2.^4^

Susceptibility to SARS appears to be primarily dependent on the affinity of the spike RBD to bind host ACE2 in target tissues in the initial viral attachment step, and differences in the affinity of the RBD for ACE2 may determine the zoonotic host and epidemiology of spread.^5^ Protein crystal structure analysis of the SARS‐CoV RBD complexed with ACE2 demonstrated that this differential binding affinity resides in a critical receptor‐binding motif of the spike receptor protein, and in particular the composition of amino acids 442, 472, 479, 480, and 487 determine affinity for human ACE2 as opposed to other hosts.^5^

Predicted protein structural analysis of these critical five residues in the SARS‐CoV‐2 analogous to SARS‐CoV suggested an increased binding affinity to human ACE2 by SARS‐CoV‐2.^6^ A synthetic SARS‐CoV‐2 RBD was found in vitro to bind and enter cells transfected only with a human ACE2 receptor and no other known coronavirus targets such as DPP4, the target of MERS‐Co‐V.^7^ Therefore, it is rational to assume that like SARS‐CoV, SARS‐CoV‐2 uses the ACE2 receptor on target tissues as its primary mechanism of entry.

Recent work with cryo electron microscopy of ACE2 bound to a spike protein fragment of SARS‐CoV‐2^8^ suggests that the viral spike protein is a trimer, with one of the trimer RBD sites exposed to bind ACE2. Additional structure‐function studies^9^ also appear to indicate that the viral spike S protein of SARS‐CoV‐2 is highly glycosylated. The S protein is cleaved by proteases into two subunits (S1 and S2) and the S1 subunit is further divided into SA and SB domains, with the SB domain predicted to bind to human ACE2. The S2 subunit is responsible for fusion of the virus‐ACE2 complex with the cell membrane, and is highly glycosylated at evolutionarily conserved sites.^9^

It is tempting to speculate that alterations in glycosylation of both the spike protein as well as ACE2 can modulate viral binding. It is also tempting to speculate that alterations in the highly glycosylated and evolutionarily conserved viral fusion subunit could modulate S protein‐ACE2 complex fusion with the cell membrane and potentially attenuate human to human transmission.

The importance of ACE2 binding by SARS‐CoV2 in COVID‐19 is underscored by the observation that anosmia and dysgeusia have recently been observed in patients with COVID‐19^10^ and that ACE2 expression has recently been found to be high in the oropharynx and tongue.^11^

## 2 HYPERGLYCEMIA AND COVID‐19 INFECTION

Binding of ACE2 by SARS‐CoV‐2 in COVID‐19 also suggests that prolonged uncontrolled hyperglycemia, and not just a history of diabetes mellitus, may be important in the pathogenesis of the disease.

A known history of diabetes (DM) and ambient hyperglycemia were found to be independent risk factors for morbidity and mortality in SARS.^12^ In a follow‐up analysis of 135 patients, high fasting plasma glucose (FPG) was an independent predictor of SARS mortality.^13^ Diabetes was found in 7.4% of a cohort of hospitalized COVID‐19 patients and appeared to be a risk factor for severity of disease.^14^ A history of diabetes was associated with 22.5% of COVID‐19 intensive care unit (ICU) admissions vs 5.9% of non‐ICU admissions in one case series,^15^ and another recent ICU case series reported 14 of 24 (58%) with a history of diabetes.^16^ Mortality of COVID‐19 in patients with diabetes was found to be 7.6% vs 0.9% in patients with no co‐morbidities.^17^

A possible explanation for a link between hyperglycemia and ACE2 levels in the severity of COVID‐19 disease could be explained by several clinical observations in SARS and preclinical observations in the NOD diabetic mouse. As noted above, potential changes in glycosylation of the ACE2, as well as glycosylation of the viral spike protein, both possibly induced by uncontrolled hyperglycemia, may alter both the binding of the viral spike protein to ACE2 and the degree of the immune response to the virus.

In a subset of 39 patients who had no prior diabetes, received no steroid treatment during hospitalization, and who survived SARS, FPG levels during hospitalization were found to decrease before discharge.^13^ Twenty of these 39 (51%) patients had diabetes during hospitalization,^13^ and at 3 years of follow‐up only 2 of 39 (5%) did. Autopsy examination of an unrelated individual revealed high levels of ACE2 expression in the alveolar tissue of the lung, the islet cells of the pancreas, the heart, and the kidney. This suggested a mechanism of transient hyperglycemia induced by a transient inflammation of the islet cells of the pancreas by SARS‐CoV through binding of SARS‐CoV to the ACE2 present on islet cells, resulting in a transient insulin dependent diabetes mellitus, which resolved with resolution of disease.^13^

In a study of NOD diabetic mice, ACE2 protein levels in the lung were putatively elevated when compared to control mice and returned to the control level when insulin was administered.^18^ By reducing levels of glycosylated ACE2 target in the target lung tissue by glycemic control, this could possibly reduce the number of glycosylated viral binding sites in the lung, and hence possibly ameliorate some of the inflammation and symptoms of COVID‐19 disease. This also suggests a possible paracrine loop hypothesis for COVID‐19 infection, where the virus infects the pancreas and lung, leading to hyperglycemia and upregulation of glycosylated ACE2 in the lung, and further virus binding and inflammation. Poor glycemic control could therefore make the disease more severe. In a case series of 138 COVID‐19 patients, glucocorticoid therapy was used in 44.9% of non‐ICU patients and 72.2% of ICU patients,^15^ and presumably this glucocorticoid use made hyperglycemia, and possibly clinical symptoms, more severe. A recent review of glucocorticoids in viral diseases recommended against use in COVID‐19 pneumonia and suggested that it could cause harm.^19^

It is interesting to note, that as this article was going to press, that another group has expressed a similar hypothesis.^20^

Potentially high and aberrantly glycosylated ACE2 in the lung, nasal airways, tongue, and oropharynx in uncontrolled hyperglycemia could also serve as increased SARS‐CoV‐2 viral binding sites, thus leading to a higher propensity to COVID‐19 infection and a higher disease severity.

Interestingly, high ACE2 has a protective effect in various organs.^21^ ACE2 gene expression is increased by estrogen in a mouse model ^22^ a potential protective factor for SARS‐CoV‐2 infection and pathogenicity, as men are more likely than women to both acquire COVID‐19 and have more severe disease. Recent survey studies found that ACE2 gene expression was higher in tissues of women and in younger adults, an inverse correlation to disease severity.^23^ A possible explanation for this discrepancy is that gene expression experiments cannot measure posttranslational modifications such as protein glycosylation. In the NOD diabetic mouse model, ACE2 activity in the lung did not rise and then fall with insulin administration, but the amount of ACE2 protein apparently did.^18^ This is consistent with a rise in glycosylated ACE2, as opposed to total ACE2, since antibody binding to proteins as measured by Western blot analysis could be affected by glycosylation.^24^ Therefore, it is likely that it is the amount of glycosylated ACE2 receptor, and not simply the amount of ACE2 alone, that is responsible for virus binding and fusion.

If true, this argues for better glycemic control in patients with pre‐diabetes and diabetes as a potential mechanism to slow COVID‐19 spread and reduce the severity of symptoms. Additionally, since 3.8% of the American population without a history of diabetes or pre‐diabetes has a hemoglobin A1c over 6.1% in random sampling,^25^ use of high A1c as a risk stratification for COVID‐19 could have merit.

## 3 ROLE OF ANTIBODY RESPONSE IN COVID‐19 DISEASE SEVERITY

Clinical information about the timing of the development of acute respiratory distress syndrome (ARDS) in SARS and COVID‐19 is also informative. In a series of 75 patients with SARS^26^ median time to oxygen desaturation occurred around day 4 after the onset of symptoms, median time to peak viral load occurred around day 10, and median time to peak ARDS requiring intubation occurred around day 10 to 12. In a series of 138 COVID‐19 patients^15^ median time to dyspnea from the onset of symptoms was 5 days, to hospital admission was 7 days, and to ARDS 8 days. In a series of 24 COVID‐19 patients admitted to ICUs in the Seattle area, the median time to hospital admission was 7 days from the onset of symptoms. Another case series from Wuhan suggests a median time from symptom onset to development of computed tomography (CT) scan changes consistent with COVID‐19 pneumonia to be a median of 5 days.^14^ It appears that both SARS and COVID‐19 have a similar clinical course of infection, symptom onset, dyspnea, and oxygen desaturation at approximately day 5 after symptoms in a fraction of symptomatic patients, followed by a worsening of disease to ARDS requiring intubation at day 7 to 10 after symptoms in a smaller fraction of dyspneic patients.

This appears to be coincident with a peak in viral load as measured in nasopharyngeal swabs at day 10 after symptom onset in SARS.^26^ The timing of the ARDS in SARS also corresponded to the timing of immunoglobulin G (IgG) seroconversion.^26^ Additionally, the neutralizing antibody (Nab) response to the SARS‐CoV spike protein in patients who died of SARS occurred earlier in the course of the disease (14.7 days after symptom onset) vs those who survived (20 days), and the Nab antibody titer was higher in those who died of SARS than in those survived.^27^ Notably, viral load in COVID‐19 in severe cases appears to remain high 10 days after symptom onset^28^ and it is tempting to hypothesize that a similar mechanism applies to SARS‐CoV‐2.

The study of SARS‐like coronaviruses in animal models has been of necessity difficult, in large part due to concerns in preventing a pandemic similar to the one we are currently experiencing. SARS experiments require a Biosafety Level 3 laboratory, and experiments where recombinant coronavirus constructs were produced by insertion of various novel spike protein RBD coding sequences into benign or non‐transmissible coronaviruses were specifically banned by the National Institutes of Health in 2014.^29^

However, experimental information exists on SARS infection and immune response from a Chinese macaque animal model.^30^ In this model, vaccination with a modified vaccina virus containing the full‐length SARS‐CoV spike protein, followed by infection with SARS‐CoV, induced varying degrees of diffuse alveolar damage in the majority of the experimental animals 7 days after infection. Adoptive transfer of serum from macaques vaccinated with the SARS‐CoV spike protein but not infected with SARS‐CoV was performed, and this serum induced diffuse alveolar damage at rates greater than control serum in SARS‐CoV‐infected macaques, indicating that the spike protein neutralizing antibodies were amplifying virus damage by SARS‐CoV in the lung, even at low doses of serum. High doses of serum still caused alveolar damage even if they reduced SARS‐CoV viral titers, and the damage appeared to be restricted to acutely infected animals. The action of the serum in inducing this damage also appeared to be due to driving of pulmonary macrophages excessively to a pro‐inflammatory M1 polarized state, possibly through involvement of the glycosylated Fc gamma receptor. Serum from deceased SARS patients in this study also induced this pro‐inflammatory M1 polarization as well.

The acute onset of lung inflammation in SARS resulting in ARDS appears to be tightly associated with monocyte/macrophage polarization and function.^31^ During acute infection, macrophages display a classically activated inflammatory M1 phenotype and express cytokines such as interleukin‐6 (IL‐6). High IL‐6 levels were associated with increased mortality in a series of 191 hospitalized patients with COVID‐19.^16^

It is tempting to consider that the same mechanism acts in COVID‐19 as in SARS, where an overactive macrophage M1 inflammatory response, as neutralizing antibodies to the SARS‐CoV‐2 spike protein form at day 7 to 10, results in ARDS in susceptible patients. It is also allows consideration of agents which may interfere with this overly brisk macrophage inflammatory response and perhaps influence the course of the disease, in particular those that blunt but do not completely abrogate M2 to M1 balance in macrophage polarization, as well as viral load, which in SARS appears to be temporally related to the onset of ARDS.

It is of interest that in a mouse model of asthma, macrophage M2 polarization is increased by estrogen.^32^ Additionally, estrogen also accelerates acquisition of an M2 phenotype in a monocyte cell culture model.^33^ Differences in M1 to M2 polarization may perhaps explain the partial protective effect of female sex in the pathogenesis of COVID‐19.

## 4 INHIBITION OF PROTEIN GLYCOSYLATION TO BOTH MODULATE VIRUS‐ACE2 INTERACTION AND BLUNT M1 MACROPHAGE POLARIZATION IN THE VIRAL IMMUNE RESPONSE

It is a tribute to the ingenuity and effort of the medical and scientific community that we currently have multiple agents in clinical trials to help treat COVID‐19, all of which have a rational basis. However, for an agent to have an effect in blunting the medical complications of a widespread pandemic, where hundreds of thousands of infected patients are at risk of fatal progression of disease, this agent needs to be cheap, relatively nontoxic, and rapidly scalable. Only one of the agents being considered at this point meets these criteria.

Hydroxychloroquine has been used of the treatment of rheumatoid arthritis and other autoimmune conditions for nearly 70 years. It has also been used as malaria prophylaxis and treatment for nearly 50 years. It is extremely safe and has been used widely in underdeveloped countries for these conditions, in children and in pregnant women, with little to no monitoring. While there are potential concerns for rare drug interactions with other agents that prolong the QT interval, and there are concerns with retinopathy with long term use, neither of these uncommon complications should be clinically significant if appropriate caution (an electrocardiogram [EKG] before therapy) is employed.^34^

There is a possible rational basis for the development of hydroxychloroquine in the treatment of COVID‐19 in clinical trials of treatment and prophylaxis. Hydroxychloroquine inhibits SARS‐CoV‐2 in vitro at concentrations achievable in human lung tissue.^35^ Chloroquine, a related compound, inhibits SARS‐CoV replication and spread in cell culture, possibly through reducing glycosylation of the ACE2 receptor.^36^ Interestingly, computer simulations suggest that hydroxychloroquine and chloroquine are predicted to bind to the active site of the enzyme UDP‐*N*‐acetylglucosamine 2‐epimerase, which catalyzes the rate determining step in the sialic acid biosynthesis pathway,^37^ and thus there is a rational basis to assume hydroxychloroquine can interfere in terminal glycosylation of proteins in the Golgi apparatus.

In a small unrandomized clinical cohort of 80 patients the combination of hydroxychloroquine and azithromycin has been shown to rapidly reduce viral load in COVID‐19 patients and ameliorate clinical symptoms in the majority of patients.^38^ In another small randomized unpublished study of 62 COVID‐19 patients, undertaken after the observation that none of a cohort 80 lupus patients in Wuhan on chronic hydroxychloroquine therapy developed COVID‐19 infection, 62 patients with mild COVID‐19 symptoms and signs of COVID‐19 pneumonia on CT scan were randomized to 200mg of hydroxychloroquine orally twice daily for 5 days and usual care or usual care alone.^39^ In the hydroxychloroquine arm of this study, 80.6% of subjects had improvement of COVID‐19 pneumonia findings on CT scan vs 50.8% of controls after 5 days of therapy (*P*=.0476) and 0% of patients on hydroxychloroquine progressed to severe disease vs 12.9% of control patients (*P*=not done).

Hydroxychloroquine also can act as an oral hypoglycemic agent, as patients with diabetes taking hydroxychloroquine for rheumatologic diseases had a significant reduction in hemoglobin A1c when compared to methotrexate,^40^ and thus can serve to reduce hyperglycemia, a possible COVID‐19 risk factor for disease severity.

While studies are mixed on this topic, and the balance of M1 to M2 macrophage polarization may differ depending on the local microenvironment, hydroxychloroquine has been shown in at least one study to block the polarization of macrophages to an M1 inflammatory subtype,^41^ and it is predicted to interfere with glycosylation of a number of proteins involved in the humoral immune response, possibly including the macrophage FcR gamma IgG receptor and other immunomodulatory proteins, potentially through inhibition of UDP‐*N*‐acetylglucosamine 2‐epimerase. In combination with potential other immunomodulatory effects, this could possibly blunt the progression of COVID‐19 pneumonia all to way up to ARDS where a potential over‐conversion to an inflammatory M1 macrophage subtype occurs in response to a postulated brisk humoral immune response to the SARS‐CoV‐2 spike protein around day 8 to 10 after symptom onset.

## 5 CONCLUSION AND SUMMARY

While we await larger randomized studies and more analysis of patient subtypes to determine who, if anyone, will benefit from hydroxychloroquine, in the context of this theoretical framework for COVID‐19 infection the results to date are supportive, although much caution should be used in interpretation of these early small clinical trials.

Regardless of the results of larger clinical trials of hydroxychloroquine in COVID‐19, it is hoped that the above analysis can provide a testable theoretical framework to allow for advances in our understanding and control of this deadly viral epidemic.

## CONFLICT OF INTERESTS

The authors declare that there are no conflict of interests.
